# Assessment of nematicidal and plant growth-promoting effects of *Burkholderia* sp. JB-2 in root-knot nematode-infested soil

**DOI:** 10.3389/fpls.2023.1216031

**Published:** 2023-07-19

**Authors:** Jong-Hoon Kim, Byeong-Min Lee, Min-Kyoung Kang, Dong-Jin Park, In-Soo Choi, Ho-Yong Park, Chi-Hwan Lim, Kwang-Hee Son

**Affiliations:** ^1^ Microbiome Convergence Research Center, Korea Research Institute of Bioscience and Biotechnology, Daejeon, Republic of Korea; ^2^ Department of Bio-Environmental Chemistry, College of Agriculture and Life Science, Chungnam National University, Daejeon, Republic of Korea; ^3^ Nematode Research Center, Life and Industry Convergence Research Institute, Pusan National University, Miryang, Republic of Korea

**Keywords:** root-knot nematode, nematicidal activity, biological control, *Burkholderia*, plant growth, *Solanum lycopersicum*

## Abstract

Root-knot nematodes (RKN), *Meloidogyne* spp., are plant-parasitic nematodes that are responsible for considerable economic losses worldwide, because of the damage they cause to numerous plant species and the inadequate biological agents available to combat them. Therefore, developing novel and eco-friendly nematicides is necessary. In the present study, *Burkholderia* sp. JB-2, isolated from RKN-infested rhizosphere soil in South Korea, was evaluated to determine its nematicidal and plant growth-promoting effects under *in vitro* and *in vivo* conditions. Cell-free filtrates of the JB-2 strain showed high levels of nematicidal activity against second-stage juveniles (J2) of *M*. *incognita*, with 87.5% mortality following two days of treatment. In addition, the assessment of the activity against other six plant parasitic nematodes (*M*. *javanica*, *M*. *hapla*, *M*. *arenaria*, *Ditylenchus destructor*, *Aphelenchoides subtenuis*, and *Heterodera trifolii*) showed that the cell-free filtrates have a broad nematicidal spectrum. The three defense-responsive (*MiMIF*-*2*, *MiDaf16*-*like1*, and *MiSkn1*-*like1*) genes were activated, while *Mi*-*cm*-*3* was downregulated when treated with cell-free filtrates of JB-2 cultures on J2. The greenhouse experiments suggested that the cell-free filtrates of the JB-2 strain efficiently controlled the nematode population in soil and egg mass formations of *M*. *incognita* in tomato (*Solanum lycopersicum* L., cv. Rutgers). An improvement in the host plant growth was observed, in which the shoot length and fresh weights of shoots and roots increased. The treatment with 10% of JB-2 cell-free filtrates significantly upregulated the expression levels of plant defenses (*SlPR1*, *SlPR5*, and *SlPAL*) and growth-promoting (*ACO1*, *Exp18*, and *SlIAA1*) genes compared with the corresponding parameters of the control group. Therefore, JB-2 could be a promising candidate for the sustainable management of RKN.

## 1 Introduction

Root-knot nematodes (RKN), *Meloidogyne* spp., are one of the most economically damaging genera of plant-parasitic nematodes, with an estimated economic loss over 100 billion USD annually (Wang et al., 2021). They are obligate endoparasites, which can infect over 3,000 plant species worldwide by invading root cells (Abad et al., 2003; Anwar and McKenry, 2010; Mitiku, 2018). The RKN J2 migrates through intercellular spaces, becomes sedentary, and enters vascular cylinders. Multinucleated feeding cells are then formed, which transform into enlarged giant cells with special nutrient-feeding structures by the end of the sedentary life cycle. It destroys the structure of host roots by depriving it of nutrients, resulting in stunted growth and production. In turn, the host gets susceptible to biotic and abiotic stresses (Ralmi et al., 2016; Liu et al., 2022). Here, the inhabitation and parasitism of RKN can be difficult to control, as its symptoms can be easily confused with other plant problems hence, identifying and controlling the occurrence of RKN in plants remain an ongoing challenge (Gillet et al., 2017; Subedi et al., 2020).

Generally, managing RKN involves the application of synthetic nematicides, botanical essential oils, biological control, host-delivered RNAi approach and resistant cultivars among others (Collange et al., 2011; Joshi et al., 2020; Subedi et al., 2020). Specifically, synthetic nematicides such as organophosphates, carbamates and fumigants have been extensively used for their efficiency; however, they are currently being restricted, owing to their negative environmental impacts such as non-target toxicity and agricultural ecosystem disturbance, as well as human health safety concerns (Forghani and Hajihassani, 2020). In the past decades, studies have been conducted to develop novel eco-friendly nematicides and address the negative impacts of the synthetic ones. Recently, biological control agents based on microorganisms have been used as more environment-friendly alternatives and are considered a sustainable nematode control strategy (Abd-Elgawad and Askary, 2018). Fungi, such as *Purpureocillium lilacinum* (e.g. BIOACT® by Bayer Crop Science), *Trichoderma* spp. (e.g. Trifesol® by Biocultivos Agricultura Sostenible) and *Pochonia chlamydosporia* (e.g. KlamiC® by BiotorLabs) has been widely accepted and utilized by farmers and other stakeholders for controlling nematode populations in soil (Moliszewska et al., 2022). Certain bacterial species, including *Bacillus* spp. (e.g. Aveo®EG by Valent BioSciences) and *Pasteuria* spp. (e.g. ClarivaTM by Syngenta International AG) are also known to be safe and cost-effective and are well-received to control a wide range of nematode species (Roth et al., 2020; Machado, 2022).

The rhizosphere environment harbors diverse bacteria that affect the soil ecosystem, promote plant growth, improve the plant defense system and exert direct antagonistic effects on plant pathogens. Particularly, certain strains of rhizosphere bacteria such as *Bacillus*, *Pseudomonas*, *Pasteuria*, *Serratia* and *Burkholderia* have been reported to play a crucial role in controlling RKN through their nematicidal and plant growth-promoting properties (Collange et al., 2011; Mhatre et al., 2019; Ahmad et al., 2021). They have been reported to effectively suppress RKN by directly paralyzing, killing and inhibiting J2 hatching through their toxic metabolite and enzyme production capabilities, in addition to having indirect suppressing abilities through the induction of systemic plant resistance (Siddiqui and Mahmood, 1999; Tian et al., 2007; Lamovšek et al., 2013). However, despite continuous research and notable success in laboratory studies of bacterial nematicides, their mechanisms in field conditions remain poorly understood; hence, extensive research, particularly on novel nematicidal strains with high field adaptability and activity in the rhizosphere, remains necessary and crucial for the development of sustainable RKN control strategies.

In the present study, the nematicidal strain *Burkholderia* sp. JB-2 was isolated from RKN-infested rhizosphere soil in South Korea, in which its ability to control RKN was assessed under laboratory conditions. Furthermore, changes in the relative expression levels of seven *M*. *incognita* J2 genes were studied. Potted tomato (*Solanum lycopersicum* L. cv. Rutgers; susceptible control) (Kokalis-Burelle et al., 2013) in soil infested with *M. incognita* under greenhouse conditions was also monitored to assess whether the JB-2 can control and promote plant growth.

## 2 Materials and methods

### 2.1 Nematodes

*Meloidogyne incognita* was collected and identified based on the method described by Saeki et al. (2003) from roots of the oriental melon (*Cucumis melo* L. var. *makuwa*) in a commercial greenhouse at Yesan-ri, Seongju-gun, Republic of Korea, which were then grown on tomato (*Solanum lycopersicum* L., cv. Rutgers, Seedway, Hall, NY) 28 ± 2°C, under greenhouse conditions.

The egg masses were obtained from infected *S*. *lycopersicum* using a 0.5% NaOCl solution according to Hussey and Barker (1973) and incubated at 28°C for 24 h in distilled water. *Meloidogyne incognita* J2 were obtained using a modified Baermann funnel (Viglierchio and Schmitt, 1983) and used for *in vitro* and *in vivo* experiments. Other pure cultured-nematodes such as *M*. *javanica, M*. *hapla, M*. *arenaria*, *Ditylenchus destructor*, *Aphelenchoides subtenuis* and *Heterodera trifolii* were obtained from the Nematode Research Center, Life and Industry Convergence Research Institute, Pusan National University, Miryang-si, Republic of Korea for the activity spectrum analysis.

### 2.2 Isolation and identification of bacterial strains

Rhizosphere soil samples were collected from a *M*. *incognita*-infested commercial greenhouse in Seongju-gun, Republic of Korea (35°55*’*32.2″ N, 128°17*’*13.8″ E). Thereafter, one gram of the collected soil samples were diluted with phosphate-buffered saline (0.8% NaCl, 0.02% KCl, 0.144% Na_2_HPO_4_, 0.024% KH_2_PO_4_, pH 7.4). The soil suspension was subsequently spread onto Reasoner’s 2A agar medium (MBcell, Seoul, Republic of Korea) and incubated at 30°C for two days. The bacterial colonies were isolated according to color and morphological properties and stored at −70°C in R2A broth with 25% sterilized glycerol for further analysis. A total of 28 bacterial isolates from rhizosphere soil samples were evaluated for nematicidal activity against *M*. *incognita* J2.

Genomic DNA was extracted using a standard phenol-chloroform extraction method (Wilson, 2001) and a partial 16S rRNA gene was amplified using a polymerase chain reaction (PCR) to identify the molecular characteristics of the bacterial isolates, in which the universal primers 27F (5′-AGAGTTTGATCMTGGCTCA-3′) and 1492R (5’-TACGGYTACCTTGTTACGACTT-3′) were used (Weisburg et al., 1991). The sequencing of the purified products was performed at Macrogen Inc. (Seoul, Republic of Korea). The sequence of the 16S rRNA gene was then evaluated against the type strains available in the EzBioCloud database (ChunLab Inc., Seoul, Republic of Korea) to identify closely related species. Molecular phylogeny of 16S rRNA was inferred using the neighbor-joining method in MEGA X software (Kumar et al., 2018).

### 2.3 *In vitro* nematicidal activities of cell-free filtrates

Bacterial isolates were fermented in a 500 mL baffled Erlenmeyer flask containing 100 mL of Luria-Bertani (LB) broth (BD Difco, Franklin Lakes, NJ, United States) on a shaking incubator (200 rpm) at 28°C for 48 h. Following fermentation (Approximately equivalent to 3.0 × 10^8^ colony-forming unit/mL), supernatants were separated by centrifugation at 10,000 rpm for 15 min at 4°C, and subsequently filtered using a 0.22 μm pore filter (Millipore, Burlington, MA, USA). An aliquot containing 50 fresh hatched-J2 in 90 μL sterilized water was transferred to each well of a 96-well plate (SPL Life Sciences Co. Ltd., Gyeonggi-do, Korea) and treated with 10 μL of cell-free filtrates at a final concentration of 10% (v/v). LB broth was used as the negative control. Abamectin (1 μg/mL, Supelco, Bellefonate, PA, USA) and 2,000-fold diluted Sunchungtan 150 EC (150 μg/mL of fosthiazate, Farm Hannong Co., Seoul, Korea) was used as the positive control. The 96-well plates were incubated at 28°C for 48 h. Following incubation, the J2, in each treatment, were observed using a stereo microscope (Olympus SZ61, Olympus Corporation, Tokyo, Japan). They were considered dead when they exhibited a straight form and immobility following stimulations using a fine needle. The relative mortality rate was calculated based on the Abott (1925) formula: [(mortality rate on the treatment-mortality rate on the negative control)/(1-mortality rate on the negative control)]. All experiments were performed in triplicate wells and repeated three times under similar conditions.

### 2.4 Total RNA isolation and cDNA synthesis

Following 24 h of treatment, total RNA was extracted from J2 using TRIzol reagent solution (Ambion, Carlsbad, CA, USA), which was then subjected to purification using the RNeasy Mini Kit (Qiagen, Hilden, Germany) according to the manufacturer’s instructions. The complementary DNA was synthesized using a cDNA synthesis kit (Thermo Fisher Scientific Baltics, Vilnius, Lithuania) and was used as a template.

### 2.5 Gene expression analysis

The relative expression levels of six *M*. *incognita* genes (response to oxidative stress, *MiMIF*-*2*, *MiDaf16-like1*, and *MiSkn1-like1*; nematode development, *Mi*-*Cpl*-*1* and *Mi-SER-1*; nematode parasitism, *Mi*-*cm*-*3*) which were treated with 10% culture filtrates of JB-2 and investigated by real-time quantitative PCR. The expression levels were analyzed using SYBR Green Master Mix (Roche Diagnostics, Mannheim, Germany) and gene-specific primers (**Supplementary Table S1**) on a 7500 Real-Time PCR system (Applied Biosystems, Foster City, CA, USA). The 18S rRNA and *MiActin* were used as the reference genes. The relative transcription levels were calculated using the 2^-ΔΔCt^ method (Beaubois et al., 2007). All experiments were performed in triplicate.

### 2.6 Reactive oxygen species assay

The fluorescent probe, 2,7-dichlorodihydrofluorescein-diacetate (H_2_DCF-DA, Sigma-Aldrich, St Louis, MO, USA), was used to detect intracellular reactive oxygen species (ROS) levels in *M. incognita* (Yoon et al., 2018; Maleita et al., 2022). The J2 of *M. incognita* were collected in sterilized water and 50 nematodes were transferred, per well, into 96-well plates. The 10% cell-free filtrates, 50 mM H_2_O_2_ (Daejung Chemicals & Metals Co., Ltd, Gyeonggi-do, Republic of Korea; as positive control), LB broth (negative control), 1 μg/mL abamectin and 2,000-fold diluted sunchungtan 150EC (150 μg/mL of fosthiazate) were poured in each well, respectively, and were subsequently incubated for 24 h at 25°C. Thereafter, the nematodes were washed thrice with sterilized water and transferred to the 96-well plate. The H_2_DCF-DA was then added to each well at a final concentration of 50 μM. The fluorescence was measured using a fluorescence spectrophotometer (Wallac Victor 3 1420 multilabel counter, Perkin-Elmer, Wellesley, USA) by recording the fluorescence intensity at λex 485 nm and λem 535 nm, at 60 min intervals for 120 min at 20°C. The data were collected from the 60 min time point. Assays were performed in triplicate wells and repeated three times under similar conditions. The nematodes were immobilized on a glass slide using 10 mM sodium azide. Images were captured using a fluorescence stereo microscope (Olympus SZX16, Tokyo, Japan) equipped with a TUCSEN Dhyana 400 DC digital camera (Olympus).

### 2.7 Greenhouse experiment

The experiment was performed in a controlled greenhouse located in the Nematode Research Center, Life and Industry Convergence Research Institute, Pusan National University, Miryang-si, Republic of Korea, under the following conditions: the temperature range was 25 ± 3°C and relative humidity was 70%, with a 12 h light/12 h dark cycle. Pots with a diameter and depth of 12 cm and 10 cm, respectively, were filled with 500 g of autoclave-sterilized soil (121°C for 1 hour) obtained from a commercial greenhouse in Seongju-gun, Republic of Korea (35°55*’*32.2″ N, 128°17*’*13.8″ E) and inoculated with *M*. *incognita* (1 J2/g of soil) in 1 mL of sterilized water. Cell-free filtrates of JB-2 were similarly prepared as described above and were used for *in vivo* assays. Following 24 h of inoculation, the experiment involved four treatments: (1) cell-free filtrates of JB-2 (10%, 1%, and 0.1%), (2) 1.8% abamectin (Sun Moon Green Science Co. Ltd., Seoul, Korea) as a positive control, (3) 2,000-fold diluted sunchungtan 150EC (150 μg/mL of fosthiazate, Farm Hannong Co) as a positive control, and (4) LB broth as a negative control.

The experiments were divided into three groups. The first group was an evaluation of the effects of four treatments on nematodes in 500 g of soil from each replicate pot, one week after treatment. The number of nematodes was determined based on the method described by Coolen (1979) under a stereo microscope (Olympus SZ61). The second and third groups consisted of *S*. *lycopersicum* at the two-leaf stage, which were transplanted into pots (one for each pot) one week after treatment. The nematode population density in *S*. *lycopersicum* roots was determined by extracting J2 from 1 g of each *S*. *lycopersicum* roots at 7 days post-transplant. Meanwhile, the latter involved assessing the 45-day post-transplant plant growth parameters such as the shoot length of the plant and fresh weight of the roots and shoots, expression levels of plant defense genes, and nematode parameters such as the number of egg masses on each tomato roots. The number of egg masses was determined using phloxine B staining (Southey, 1986).

Finally, grounded root samples (0.5 g) were frozen in liquid nitrogen. The expression levels of the plant defense (salicylic acid response, *SlPR1* and *SlPR5*; salicylic acid biosynthesis, *SlPAL*) and growth-promoting (*ACO1*, *Exp18*, and *SlIAA1*) genes, were determined using gene-specific primers. The *Ubi3* and *SlActin* genes were used as the reference genes (**Supplementary Table S2**). All experiments were performed in triplicate.

### 2.8 Statistical analyses

One-way ANOVA was performed using SPSS software (version 24, SPSS, Inc., Chicago, IL, United States). The mean values were compared using Scheffé’s method and *p* values <0.05 were considered statistically significant.

## 3 Results

### 3.1 Isolation and identification of bacterial strain with nematicidal activity

**Figure 1:**
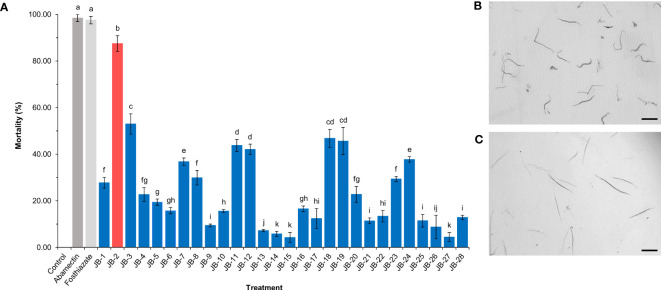
Isolation and screening of *Burkholderia* sp. JB-2 with nematicidal activity against the second-stage juveniles of *Meloidogyne incognita*. The mortality rate of the 50 fresh hatched-J2 of *M*. *incognita* after 48 h treated with 10% cell-free filtrates of isolated bacteria **(A)**. Abamectin (1 μg/mL) and 2,000-fold diluted Sunchungtan 150EC (150 μg/mL of fosthiazate) used as the positive controls, a LB broth used as the negative control. Morphological observation of the active nematodes **(B)** and the dead nematodes with straight form and immobility **(C)** post-stimulation using a fine needle. The experiment was performed in triplicate under the same conditions. Different letters above the error bars indicate significant differences by Scheffé’s test (*P* < 0.05). Scale bar: 100 μm.

Among the isolated strains, cell-free filtrates of the JB-2 strain showed high levels of nematicidal activity against J2, with a mortality rate of 87.46% (**Figure 1**). Meanwhile, the mortality rates of J2 in the positive controls of the 150 μg/mL fosthiazate and 1 μg/mL abamectin were 97.56% and 98.43%, respectively. Hence, the JB-2 strain exhibited a high nematicidal activity against J2 of *M*. *incognita* and was selected for the further studies.

The assessment of the activity spectrum using the bioassay showed broad-spectrum activities of the seven plant parasitic nematodes used *M*. *incognita*, *M*. *javanica*, *M*. *hapla*, *M*. *arenaria*, *D. destructor*, *A. subtenuis* and *H. trifolii* with a mortality rate of 87.46%, 84.36%, 83.19%, 82.83%, 81.33%, 79.28% and 74.05%, respectively (**Table 1**).

**Table 1:** Assessments of the mortality rate (%) of cell-free filtrates of *Burkholderia* sp. JB-2 against the second-stage juveniles of seven plant parasitic nematodes.

Plant parasitic nematode	Mortality rate (%)		
	Control	Fosthiazate (150 μg/mL)	JB-2 (10% cell-free filtrates)
***Meloidogyne incognita***	0.00 ± 0.00^c^	97.56 ± 1.85^a^	87.46 ± 1.09^b^
***Meloidogyne javanica***	0.00 ± 0.00^c^	96.02 ± 0.73^a^	84.36 ± 0.55^b^
***Meloidogyne hapla***	0.00 ± 0.00^c^	97.63 ± 1.00^a^	83.19 ± 0.74^b^
***Meloidogyne arenaria***	0.00 ± 0.00^c^	96.19 ± 0.96^a^	82.83 ± 0.66^b^
***Ditylenchus destructor***	0.00 ± 0.00^c^	88.41 ± 3.29^a^	81.33 ± 1.28^b^
***Aphelenchoides subtenuis***	0.00 ± 0.00^c^	90.11 ± 0.32^a^	79.28 ± 0.67^b^
***Heterodera trifolii***	0.00 ± 0.00^c^	95.08 ± 0.77^a^	74.05 ± 4.29^b^

Within a row, values with different letters indicate significant differences by Scheffé’s test at P < 0.05. Data are presented as Mean ± SD (n =3).

**Figure 2:**
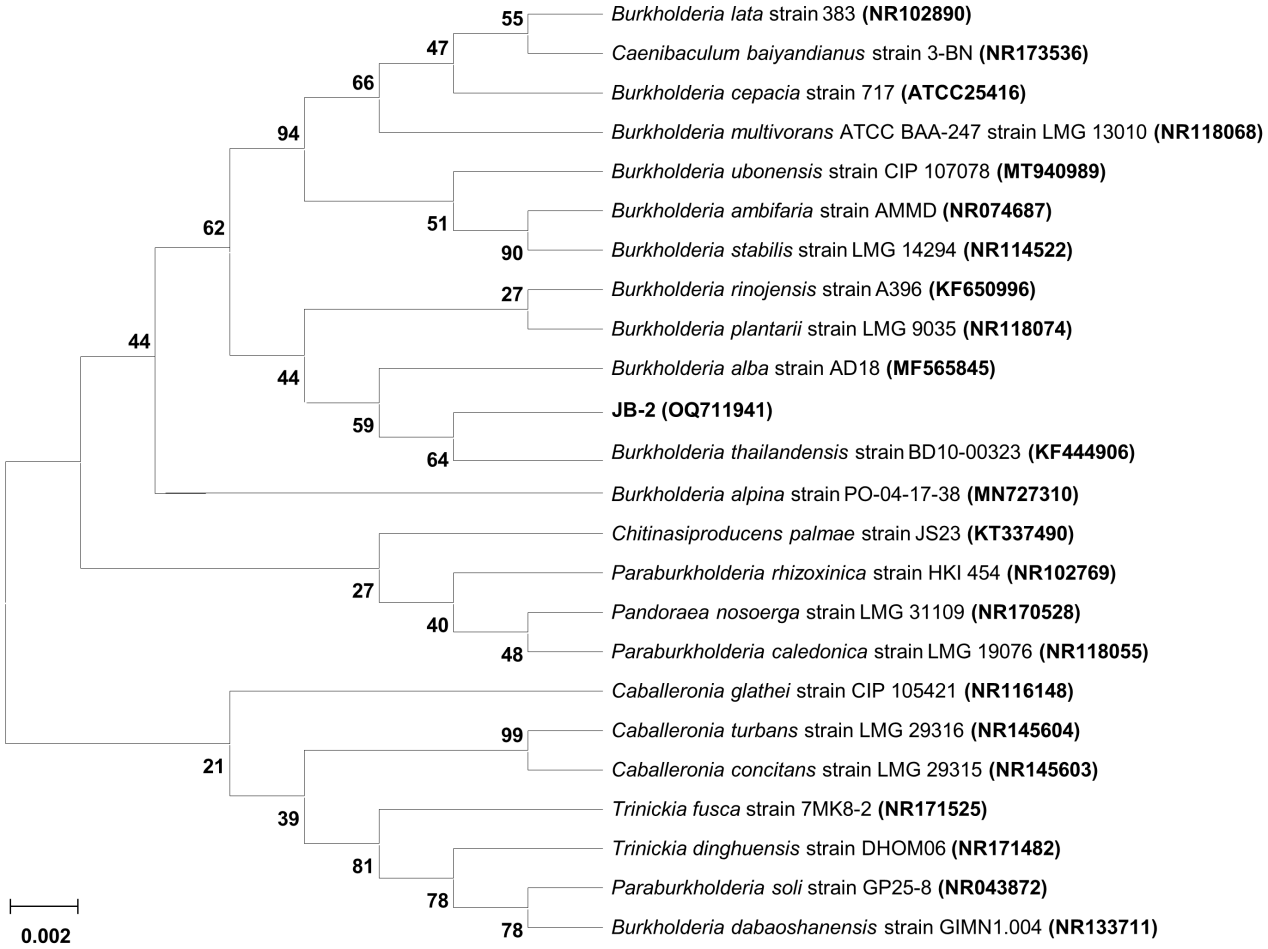
Phylogenetic relationship of the *Burkholderia* sp. JB-2 based on 16S rRNA gene sequence. Neighbor-joining phylogenetic tree based on 16S rRNA gene sequences and closely related species constructed using MEGA X software. Numbers at each branches indicate the bootstrap percentage of 1,000 replications.

The phylogenetic profiling of the JB-2 strain based on comparison between the nucleotide sequence of its partial 16S rRNA gene with that of the type strains available in the EzBioCloud database showed that JB-2 strain was most closely related to *B*. *thailandensis* BD10-00323 (GenBank accession number KF444906), in which 99.04% of the 16S rRNA nucleotide sequence showed similarities (**Figure 2**). The 16S rRNA nucleotide sequence was deposited in GenBank under accession number OQ711941. With this, the JB-2 strain was deposited in the Korean Collection for Type Cultures under code number KCTC14976BP.

### 3.2 Changes in the response properties of *M*. *incognita* upon JB-2 treatment

**Figure 3:**
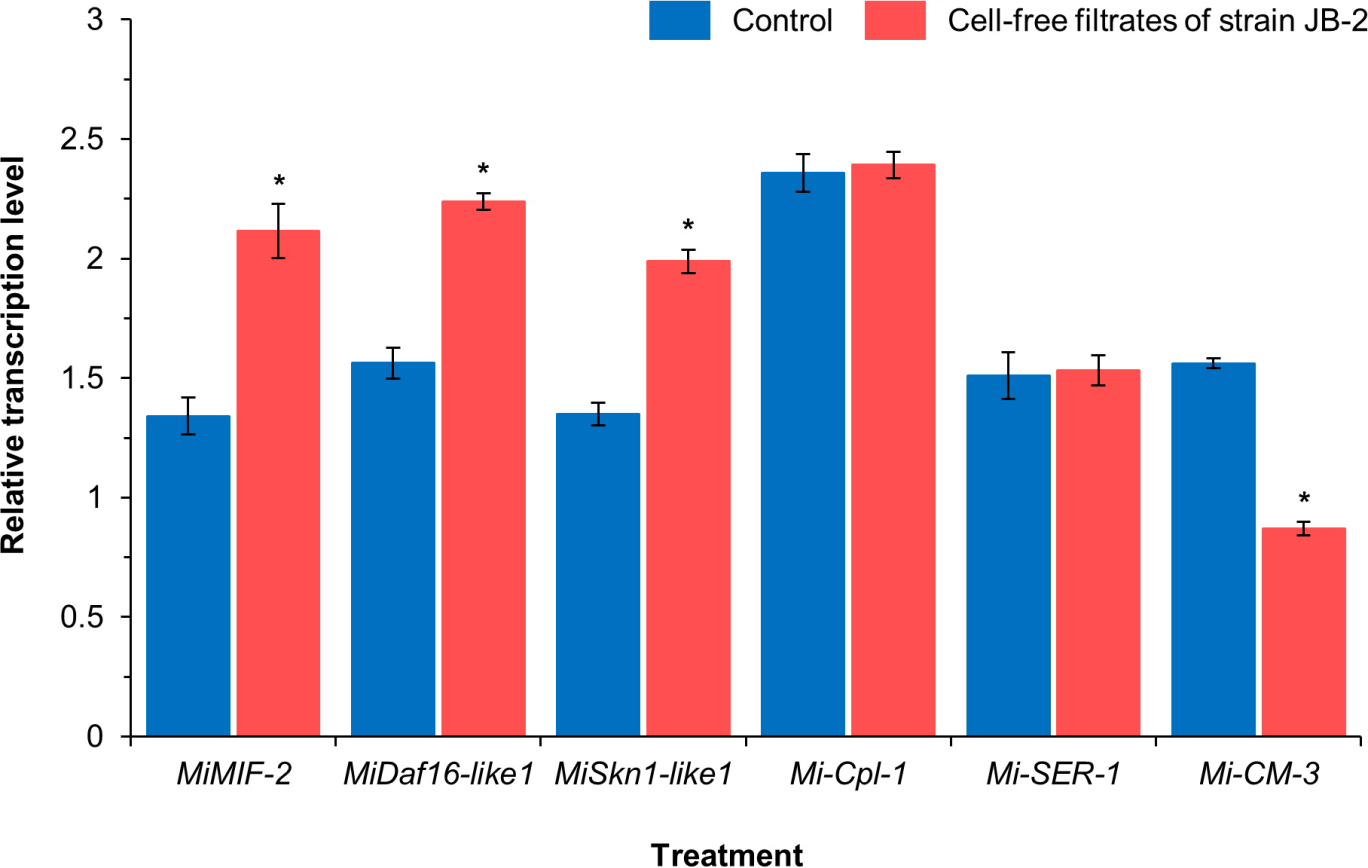
Expression profiles of the six *Meloidoyne incognita* genes upon treating with the cell-free filtrates of *Burkholderia* sp. JB-2. Data are presents as Mean ± SD (*n* = 3). Statistical significance between compared groups are indicated as *P < 0.05.

Based on the relative transcription levels of the six *M*. *incognita* genes using real-time quantitative PCR, the expression profiles of the four genes were significantly different between the control and JB-2 treatment groups (**Figure 3**), in which the expression levels of the former, including *MiMIF*-*2* (*P* < 0.005), *MiDaf16*-*like1* (*P* < 0.005), and *MiSkn1*-*like1* (*P* < 0.005), and defense-responsive genes from oxidative stress were higher by approximately 1.4 folds than that of the control. The relative expression of *Mi*-*cm*-*3*, which is a negative regulator of the salicylic acid pathway, was also reduced by 1.8-fold in treated nematodes compared with that of the control. Meanwhile, no significant differences were found between the expression levels of *Mi*-*Cpl*-*1* and *Mi-SER-1* between the control and JB-2 treatments (*P* > 0.05).

**Figure 4:**
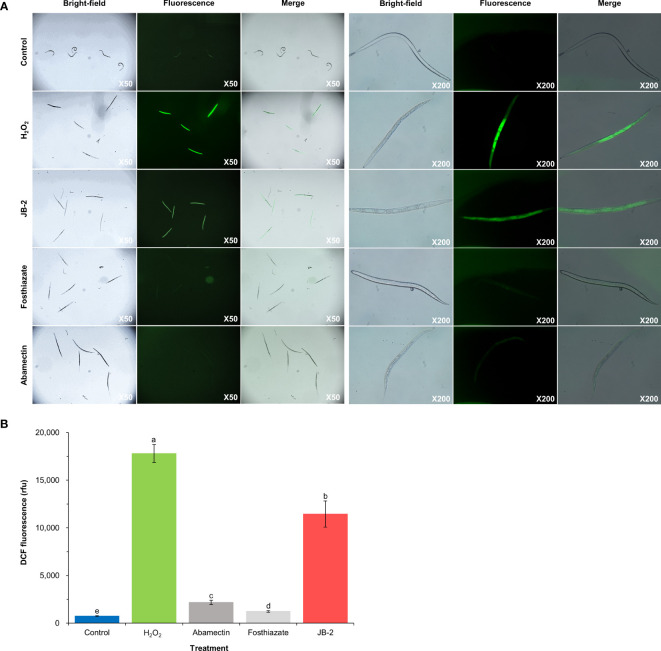
Effect of *Burkholderia* sp. JB-2 cell-free filtrates on ROS accumulation in *Meloidogyne incognita* J2. The 50 J2 of *M. incognita* were treated with 10% cell-free filtrates of JB-2 and were subsequently incubated for 24h at 25°C. 50 mM H_2_O_2_ was used as the positive control and LB broth was used as the negative control. Quantitative analysis of vacuolization and ROS accumulation in *M*. *incognita* using a fluorescence probe, H_2_DCF-DA **(A)**. Left-hand panels are bright-field images; right-hand panels are fluorescent images of the nematodes captured by the fluorescence stereo microscope equipped with a TUCSEN Dhyana 400 DC digital camera. Qualitative analysis of ROS accumulation in *M*. *incognita* J2 using a fluorescence spectrophotometer by recording the fluorescence intensity at λex 485 nm and λem 535 nm **(B)**. The experiment was performed in triplicate under the same conditions. Different letters above the error bars indicate significant differences by Scheffé’s test (*P* < 0.05).

The measurement of the intracellular ROS levels in J2 using H_2_DCF-DA (**Figure 4**) showed that, in the JB 2 treatment, there were numerous giant vacuole formations and high levels of DCF fluorescence, with a value of 11,445 ± 934 rfu, which was similar to that of the pattern observed in the H_2_O_2_-treated group. Meanwhile, the LB broth, abamectin and fosthiazate-treated groups exhibited less vacuole formation and lower levels of DCF fluorescence, with 740 ± 34, 2176 ± 210 and 1232 ± 934 rfu, respectively. These results indicate that the treatment with the JB-2 cell-free filtrate results in an increase in ROS accumulation in *M*. *incognita*, which may consequently cause oxidative stress and potential apoptosis in nematodes.

### 3.3 Effect of JB-2 strain on *M*. *incognita* under greenhouse conditions

**Figure 5:**
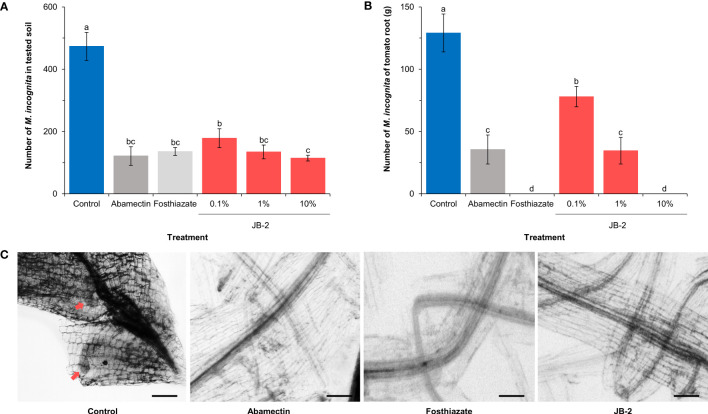
Effect of *Burkholderia* sp. JB-2 cell-free filtrates on *Meloidogyne incognita* in the pot experiment (*n* = 5). **(A)** Number of nematodes in the 500 g of soil tested. **(B)** Number of nematodes per gram of *Solanum lycopersicum* roots. **(C)** Images of *S. lycopersicum* roots at 7-day post-transplant. The arrows indicate live *M*. *incognita*. The experiment was performed in triplicate under the same conditions. Different letters above the error bars indicate significant differences by Scheffé’s test (*P* < 0.05). Scale bars: 200 μm.

Based on the week-long treatment observation of the population of *M*. *incognita* in the tested soil and *S. lycopersicum* roots (**Figure 5**), the nematode population in the soil treated with JB-2 cell-free filtrates was found to be significantly reduced (*P* < 0.005) by up to 75.9% compared to of the control. Meanwhile, no statistically significant differences were found between JB-2 and the positive control (fosthiazate and abamectin) treatment (*P* > 0.05) (**Figure 5A**). The number of nematodes per gram of *S. lycopersicum* root also significantly decreased in a concentration-dependent condition compared to the control when treated with cell-free filtrates of JB-2 (**Figures 5B, C**).

**Figure 6:**
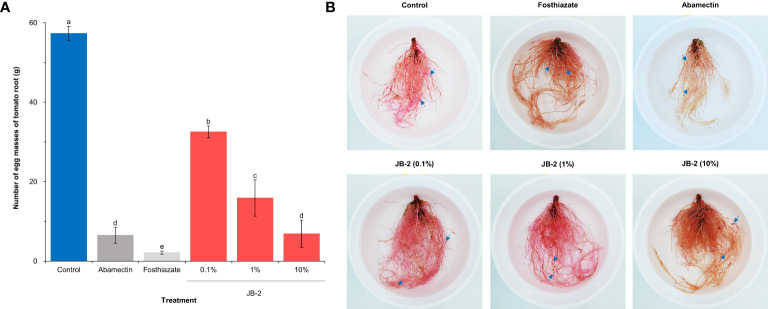
Effects of *Burkholderia* sp. JB-2 cell-free filtrates on the number of egg masses of *Meloidogyne incognita* per plant in the pot experiment (*n* = 5). **(A)** Number of egg masses on each *Solanum lycopersicum* root following 45 days of transplanting in the 500 g of the soil tested. **(B)** Root symptoms of *S*. *lycopersicum*. The arrows indicate egg masses of *M*. *incognita*. The experiment was performed in triplicate under the same conditions. Different letters above the error bars indicate significant differences by Scheffé’s test (*P* < 0.05).

Based on the number of egg masses on each *S. lycopersicum* root following 45 days of transplanting (**Figure 6**), it was found that the number of egg masses decreased in varying degrees in all treatments. Specifically, the number of egg masses (6.8 ± 3.4 egg masses/g) in the 10% cell-free filtrates of the JB-2 strain was reduced. Meanwhile, the number of egg masses in abamectin (6.5 ± 1.9 egg masses/g), including the other concentrations was also reduced and similar to that of the positive control. Hence, this suggests that the reduction was dependent on the concentration. The treatment of foasthiazate showed the highest reduction in egg masses with 2.1 ± 0.4 egg masses/g of root.

### 3.4 Effects of JB-2 strain on *S*. *lycopersicum* infested with *M*. *incognita*

**Figure 7:**
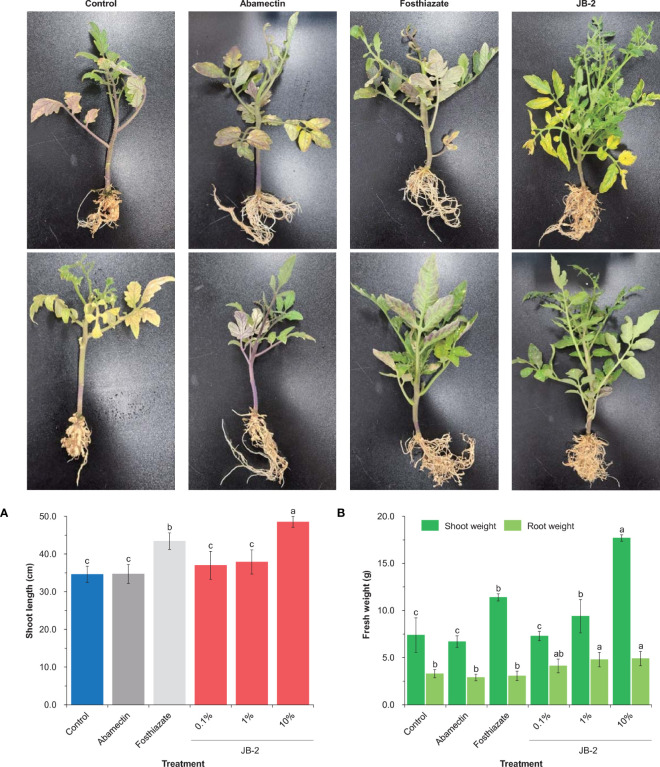
Effects of *Burkholderia* sp. JB-2 cell-free filtrates on the growth of *Solanum lycopersicum* in the pot experiment (*n* = 5). Shoot length of *S. lycopersicum* **(A)** and fresh weight of *S. lycopersicum* roots and shoots **(B)**. The experiment was performed in triplicate under the same conditions. Different letters above the error bars indicate significant differences by Scheffé’s test (*P* < 0.05).

The application of 10% cell-free filtrates of the JB-2 strain was found to significantly affect all plant growth parameters following 45 days of treatment. Specifically, it was found that it had significant effect on the growth of *S*. *lycopersicum* compared to that of the positive control (abamactin and fosthiazate) (**Figure 7**), in which shoot length growth reached 48.5 ± 1.41 cm. Other treatments did not have significant differences (*P* > 0.05), except for the fosthiazate treatment which had a shoot length growth that reached 43.4 ± 3.1 cm. In addition, the fresh weight of the shoots also increased by approximately 2.4-fold and 2.6-fold compared to that of the control and abamectin treatments, respectively. However, no statistical differences were found in the fresh weight of the roots among all the groups tested.

**Figure 8:**
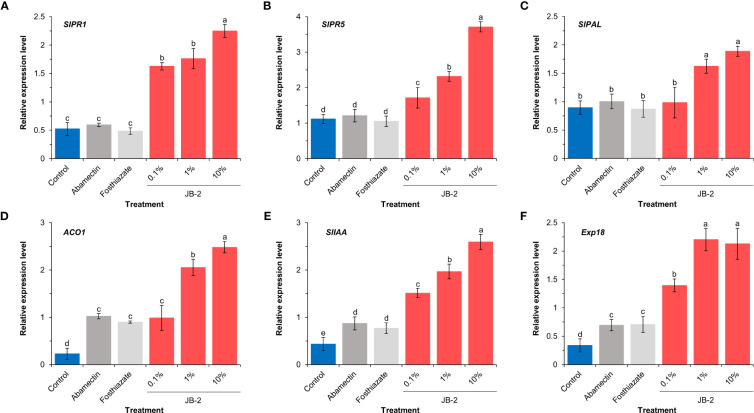
Expression profiles of genes related to defense responses **(A-C)** and plant growth **(D-F)** in tested plant roots (*n* = 5). Relative expression level of *SlPR1* **(A)**, *SlPR5* **(B)**, *SlPAL* **(C)**, *ACO1* **(D)**, *SlIAA* **(E)** and *Exp18* **(F)** in *Solanum lycopersicum* roots. The experiment was performed in triplicate under the same conditions. Different letters above the error bars indicate significant differences by Scheffé’s test (*P* < 0.05).

Based on the analysis of the expression levels of salicylic acid response (*SlPR1* and *SlPR5*), salicylic acid biosynthesis (*SlPAL*) and growth-promoting (*ACO1*, *SlIAA*, and *Exp18*) genes using real-time quantitative PCR (**Figure 8**), to determine the molecular effect of cell-free filtrates of the JB-2 strain in plants, the expression patterns of defense-related genes (*SlPR1*, *SlPR5*, *and SlPAL*) were significantly upregulated following treatment with 10% JB-2, whereas, no significant differences were found between the control and the positive control treatments (*P* > 0.05) (**Figures 8A-C**). Compared to the control, it showed more than 3-fold increases of both *SlPR1* and *SlPR5* expression and three plant growth-related genes (*ACO1*, *SlIAA*, and *Exp18*) exhibited enhanced expression levels in 10% of the JB-2 treatments by approximately 11.2-fold, 6.0-fold, and 6.4-fold, respectively (**Figures 8D-F**). Similarly, treatments with positive controls (abamectin and fosthiazate) were significantly upregulated (*P* < 0.05).

## 4 Discussion

Considering the undesirable effects of synthetic nematicides to the environment and human health, eco-friendly biological nematicides have been extensively developed over the last several decades in which biological control agents, such as numerous microorganisms, have been identified as effective alternatives that can act as nematicidal and promote plant growth (Siddiqui and Mahmood, 1999; Forghani and Hajihassani, 2020). Specifically, rhizospheric bacteria have been considered as promising alternatives, owing to their natural abundance in soils and beneficial effects to the host plant, in which they can stably colonize the rhizosphere, stimulate plant growth and development and offer host plants the essential nutrients through its dynamic interaction with the surrounding soil environment (Gray and Smith, 2005; Groover et al., 2020; Khairy et al., 2021).

Recently, *Burkholderia* species, which are gram-negative proteobacteria, widely distributed in various terrestrial and aquatic environments, have gained increasing attention as one of the most beneficial biological nematicides to control RKN, owing to their bioremediation properties, particularly of xenobiotic compounds and plant growth promotion (Coenye and Vandamme, 2003; Mahenthiralingam et al., 2005). In the present study, the 10% cell-free filtrates of *Burkholeria* sp. JB-2 had an 87% mortality rate against J2 of *M. incognita*, which also included a broad nematicidal spectrum. This indicates that the strain may possess nematicidal metabolites secretion properties; therefore, it can be considered as a suitable candidate for biological control of RKN. The results of the present study were consistent with those of previous studies, in which the nematicidal capabilities were also reported based on the effective control of *M*. *incognita* in the culture filtrates of *B*. *arboris* J211 from tobacco rhizosphere soils (Zhang et al., 2022). Similarly, it was also found that it stimulated plant growth. Meanwhile, the *B*. *vietnamiensis* B418, obtained from the barley soil and belonging to the *B*. *cepacia* complex, also exhibited nematicidal efficacy, with a 71.15% mortality rate against RKN in *Citrullus lanatus* cv. Jingxin (watermelon), in which it was found to simultaneously modulate the rhizosphere microbial community (Liu et al., 2022); additionally, the *B*. *rinojensis* A396 strain from the soils in Japan had already been commercialized as a broad-spectrum bionematicide by Marrone Bio Innovations and has been registered under the product name Majestene® (Cordova-Kreylos et al., 2013; Arthurs and Dara, 2019).

Understanding the nematicidal mechanism in controlling RKN is crucial, as they are also affected by diverse biotic and abiotic factors, in which these mechanisms largely influence their activity and stability in various environmental conditions. In turn, more effective and long-lasting RKN management can be developed to aid in identifying suitable integrated pest management strategies. However, most *Burkholderia* species have limited nematicidal mechanisms, as they are more generally associated with host defenses such as systemic acquired resistance. Additionally, its other mechanisms are yet to be identified. Macrophage migration inhibitory factor (MIF)-like proteins are multifunctional proteins that mainly regulate innate and adaptive immune responses (Leyton-Jaimes et al., 2018), for which previous research demonstrated that the MIF-like protein MiMIF-2 protected *M*. *incognita* against oxidative stress by modulating host immunity (Zhao et al., 2020). Similarly, *MiDaf16-like1* and *MiSkn1-like1* were also found to have modulating effects in response to oxidative stress, thereby activating insulin/insulin-like signaling pathways (Basso et al., 2020). In the present study, three defense responsive genes (*MiMIF-2*, *MiDaf16-like1*, and *MiSkn1-like1*) from oxidative stress were upregulated upon treatment with cell-free filtrates of JB-2 on *M*. *incognita* J2 for 24 h, indicating the activation of the defensive responses to oxidative stress. Here, the oxidative stress caused by the excessive accumulation of ROS is extensively affected by metabolic processes, leading to cell death as damage was induced to cell components (Berlett and Stadtman, 1997). The significant differences found in the fluorescence intensity and vacuole formation between the control and treatment groups indicate that there was excessive generation of ROS in nematodes, as induced by cell-free filtrates of JB-2. These results suggest that JB-2 has a direct nematicidal mechanism by inducing ROS accumulation and internal damage to *M*. *incognita*. This result was also consistent with those of Gao et al. (2016), in which the *Bacillus cereus* strain S2 was found to induce ROS accumulation in the intestinal tract and destroy the genital areas of nematodes by producing sphingosine. Three nematicidal volatiles, including dimethyl disulfide, methyl isovalerate and 2-undevanone from the *B*. *atrophaeus* strain GBSC56, also showed strong nematicidal activity, causing excessive ROS production in *M*. *incognita* (Ayaz et al., 2021). The present study is the first to demonstrate one of the prospective mechanisms of the direct nematicidal action of *Burkholderia* sp. against *M*. *incognita*. However, the exact nematicidal metabolites produced by the JB-2 strain remain unascertained. Consequently, further studies focusing on the identification of chemical properties, including the characteristics of ROS damages and its mechanisms, are necessary to validate the results. Chorismate mutase, encoded by *Mi*-*cm*-3, is an enzyme that plays an important role in the successful parasitism of *M*. *incognita* in its early parasitic stages by regulating the plant salicylic acid pathway (Wang et al., 2018). In the present study, *Mi-cm-3* was downregulated upon treatment with cell-free filtrates of JB-2 on *M*. *incognita* J2. However, the expression levels of *Mi*-*Cpl*-*1*, which encodes the cathepsin L-type cysteine protease, and Mi-*SER*-*1*, which encodes the chymotrypsin-like serine protease, did not have significant differences between the control and JB-2 treatment, despite being implicated in nematode parasitism and development (Shindo and Van der Hoorn, 2008; Antonino de Souza Júnior et al., 2013).

The results of the pot experiment indicated that despite the removal of the cells, the cell-free filtrates of JB-2 can still control *M*. *incognita*, in which plant growth-promoting effects were observed under greenhouse conditions. This also indicates that cells are not required for their activity; instead, it is inferred that secreted metabolites from JB-2 may be responsible for inducing the direct nematicidal effects and changing the soil microbiome. According to Liu et al. (2022), *B*. *vietnamiensis* B418 significantly suppresses RKN by modulating the rhizosphere microbial community, based on the changes in the composition of the soil bacterial community. Similarly, previous studies have also indicated that the changes in soil microbial communities are key factors that affect RKN colonization (Cao et al., 2022; Lu et al., 2023). It is recommended that the soil microbiome, including its microbial communities, is further studied using metatranscriptomic, metabolomic and proteomic studies to understand and verify the results of the present study.

Meanwhile, the results from the relative transcription-level analysis of the six *S. lycopersicum* genes showed that treatment with JB-2 cell-free filtrates activated the defense mechanisms of the plants. Salicyclic acid (SA) and jasmonic acid are inferred to be the major signaling molecules that regulated plant defense responses (Shoresh et al., 2005; Chen et al., 2010), in which these were induced by several endogenous phytohormones, such as SA, *SlPR1*, *SlPR5* and *SlPAL* which served as marker genes in the pathogen resistance (Seo et al., 2008; Li et al., 2015). In the present study, the relative expression levels of *SlPR1*, *SlPR5* and *SlPAL* in the *S. lycopersicum* roots had a significant increase when treated with JB-2 cell-free filtrates, consistent with the results of a previous study, which indicated that certain bacteria promoted the expression of these genes (Ayaz et al., 2021; Tian et al., 2022). *ACO1* was also suggested to be responsible for the final step in the ethylene biosynthesis pathway (Houben and Van de Poel, 2019), while *Exp18* played a crucial role in the initiation of leaf primordium (Reinhardt et al., 1998); furthermore, *SllAA*, which is involved in auxin synthesis pathway genes (Nebenführ et al., 2000), was upregulated in the 10% JB-2 treatment.

## 5 Conclusion

The JB-2 strain from the RKN-infested rhizosphere soil in South Korea displayed high levels of nematicidal activity, in which the operational mechanism involved the induction of the accumulation of excessive ROS and internal damage of the J2 of *M*. *incognita*. Under greenhouse conditions, the JB-2 strain also showed suppression of *M*. *incognita* population by reducing the number of egg masses. The strain also effectively promoted the growth of *S. lycopersicum* based on plant height, fresh weight of root and shoot, in addition to upregulating the gene expression related to plant defense and growth. However, further studies are required to determine the major factors that affect nematicidal activities, host plant and soil microbiome interactions to aid in the identification of sustainable management of RKN (*e*.*g*. host plant and nematode species, verification of mechanisms at the molecular level, related hormone and metabolites, field conditions and soil microbial communities). The findings of the present study suggest that the JB-2 strain can be considered a potential alternative to nematicides with multi-functional benefits. Therefore, these findings provide further understanding of the multiple interactions that occur among rhizospheric bacteria, RKN and plants.

## Data availability statement

The datasets presented in this study can be found in online repositories. The names of the repository/repositories and accession number(s) can be found in the article/**Supplementary Material**.

## Author contributions

J-HK, C-HL and K-HS participated in acquiring the data, the study design, drafted the manuscript, and revised the final manuscript. B-ML carried out the all of the laboratory experiments. M-KK participated in the data analyses. D-JP and H-YP participated in the protocol design and the statistical analyses. I-SC supplied the nematodes and conducted the pot trial. All the authors read and approved the manuscript.
