# Supplementary material for: Assessment of nematicidal and plant growth-promoting effects of Burkholderia sp. JB-2 in root-knot nematode-infested soil
Source: Front Plant Sci. 2023 Jul 19;14:1216031. doi: 10.3389/fpls.2023.1216031 (PMC10394650; doi:10.3389/fpls.2023.1216031)
Supplement: Supplementary file 1 [file Table_1.docx]

Supplementary Material

Assessment of nematicidal and plant growth-promoting effects of Burkholderia sp. JB-2 in root-knot nematode-infested soil

Jong-Hoon Kim, Byeong-Min Lee, Min-Kyoung Kang, Dong-Jin Park, In-Soo Choi, Ho-Yong Park, Chi-Hwan Lim^*^, Kwang-Hee Son^*^

*** Correspondence:** Chlim@cnu.ac.kr (C.-H. Lim), sonkh@kribb.re.kr (K.-H. Son)

# Table S1 Nucleotide sequences of *M*. *incognita* gene primers used in this study.

| **Genes** | | **Primer** | **Sequence (5’ to 3’)** | **Size (bp)** | **Reference** |
| --- | --- | --- | --- | --- | --- |
| *Meloidogyne incognita* | *18S* | Forward | ACCGTGGCCAGACAAACTAC | 114 | Dubreuil et al. (2011) |
|  |  | Reverse | GATCGCTAGTTGGCATCGTT |  |  |
|  | *MiActin* | Forward | GATTCGTATGTGGGAGATGAGG | 375 | Moreira et al. (2022) |
|  |  | Reverse | TTAGCCTTTGGGTTGAGAGG |  |  |
|  | *MiMIF*-*2* | Forward | ATGGTTCTAGTTAATGCTGGG | 339 | Zhao et al. (2020) |
|  |  | Reverse | GGACCAGTCAATTTCATATAAAC |  |  |
|  | *MiDaf16*-*like1* | Forward | CATGGACATTCTGCTCCCCTT | 102 | Basso et al. (2020) |
|  |  | Reverse | GGCTGTTGTTGCTGCCAAAT |  |  |
|  | *MiSkn1*-*like1* | Forward | TCCAACCACCAACAGCAACA | 84 | Basso et al. (2020) |
|  |  | Reverse | ACGTGAACGTTGCCTTGAATG |  |  |
|  | *Mi*-*Cpl*-*1* | Forward | TGTACACTTTGCTTGTCGAG | 103 | Antonino de Souza Júnior et al. (2013) |
|  |  | Reverse | GAATTTCTTCGAGATCGTTG |  |  |
|  | *Mi-SER-1* | Forward | CATTTTCCGACCTTGCACTT | 157 | Antonino de Souza Júnior et al. (2013) |
|  |  | Reverse | GGCGGTCATTGAGCAAACT |  |  |
|  | *Mi*-*cm*-*3* | Forward | GATACAGAATTCGCCCGTCT | 350 | Wang et al. (2018) |
|  |  | Reverse | TTCATTTGGCAGTCCAGTTC |  |  |
