# Supplementary material for: Assessment of nematicidal and plant growth-promoting effects of Burkholderia sp. JB-2 in root-knot nematode-infested soil
Source: Front Plant Sci. 2023 Jul 19;14:1216031. doi: 10.3389/fpls.2023.1216031 (PMC10394650; doi:10.3389/fpls.2023.1216031)
Supplement: Supplementary file 2 [file Table_2.docx]

Supplementary Material

Assessment of nematicidal and plant growth-promoting effects of Burkholderia sp. JB-2 in root-knot nematode-infested soil

Jong-Hoon Kim, Byeong-Min Lee, Min-Kyoung Kang, Dong-Jin Park, In-Soo Choi, Ho-Yong Park, Chi-Hwan Lim^*^, Kwang-Hee Son^*^

*** Correspondence:** Chlim@cnu.ac.kr (C.-H. Lim), sonkh@kribb.re.kr (K.-H. Son)

# Table S2 Nucleotide sequences of *S*. *lycopersicum* gene primers used in this study.

| **Genes** | | **Primer** | **Sequence (5’ to 3’)** | **Size (bp)** | **Reference** |
| --- | --- | --- | --- | --- | --- |
| *Solanum lycopersicum* L. | *Ubi3* | Forward | GTGTGGGCTCACCTACGTTT | 162 | Bhattarai et al. (2008) |
|  |  | Reverse | ACAATCCCAAGGGTTGTCAC |  |  |
|  | *SlActin* | Forward | ATGTATGTTGCCATCCAGGCT | 310 | Chinnapandi et al. (2017) |
|  |  | Reverse | TGTGGCTGACACGATCTCCA |  |  |
|  | *SlPR1* | Forward | CCAAGACTATTCTTGCGGTTCA | 112 | Li et al. (2015) |
|  |  | Reverse | CGCTCTTGAGTTGGCATAGT |  |  |
|  | *SlPR5* | Forward | CCCAAACACCCTAGCTGAAT | 110 | Li et al. (2015) |
|  |  | Reverse | GGGCGAAAGTCATCGGTATATTA |  |  |
|  | *SlPAL* | Forward | TGATGAACGGAAAGCCTGAA | 128 | Li et al. (2015) |
|  |  | Reverse | CTGAGCTGCCTTGACATAAGA |  |  |
|  | *ACO1* | Forward | GATCAAGGGACTCCGCGCTC | 123 | Ayaz et al. (2021) |
|  |  | Reverse | TGGCGCATGGGAGGAACATC |  |  |
|  | *SlIAA* | Forward | AAACCCCACCACCTGTTGCC | 271 | Ayaz et al. (2021) |
|  |  | Reverse | GCAGGGGCAAATTCAGAGCC |  |  |
|  | *Exp18* | Forward | GGTGGAGCGTGTGGGTATGG | 236 | Ayaz et al. (2021) |
|  |  | Reverse | GGAGGGTTACACCAACCGCC |  |  |
